# Analytical and numerical comparisons of two methods of estimation of additive × additive × additive interaction of QTL effects

**DOI:** 10.1007/s13353-021-00676-7

**Published:** 2021-12-23

**Authors:** Adrian Cyplik, Jan Bocianowski

**Affiliations:** Department of Mathematical and Statistical Methods, Poznań University of Life Sciences, Wojska Polskiego 28, 60-637 Poznań, Poland

**Keywords:** Doubled haploid (DH) lines, Barley, QTL interaction, Genetic interactions, Statistical methods

## Abstract

This paper presents the analytical and numerical comparison of two methods of estimation of additive × additive × additive (*aaa*) interaction of QTL effects. The first method takes into account only the plant phenotype, while in the second we also included genotypic information from molecular marker observation. Analysis was made on 150 doubled haploid (DH) lines of barley derived from cross Steptoe × Morex and 145 DH lines from Harrington × TR306 cross. In total, 153 sets of observation was analyzed. In most cases, *aaa* interactions were found with an exert effect on QTL. Results also show that with molecular marker observations, obtained estimators had smaller absolute values than phenotypic estimators.

## Introduction

The analysis of inheritance of quantitative traits, due to their polygenic nature, requires the use of appropriate statistical and genetic methods. Among these methods, the most interesting are those that enable the determination of the mode of action of genes in the studied population.

The concept of genetic interactions is known for more than a hundred years (Bateson and Mendel 1902). Considering that a complex phenotype may be the effect of a combination of multiple loci, various statistical methods have been developed for identifying genetic epistasis effects (Chen et al. 2011). Most studies are focused on single locus analysis, which directly tests the association between individual genes and phenotypic variants. Pairwise interactions are often used in modern genetics (Brem et al. 2005; Jarvis and Cheverud 2011; Gaertner et al. 2012), but higher-order interactions are often neglected. This kind of more complex interaction requires complete, precise data to be successfully included, but this type of data was rarely available since more recent times (Carlborg et al. 2006; Cordell 2009). There is no denying that we do not fully understand all of the mechanics of heritability and the higher-order interactions may be the missing element of explaining the relationship between genotype and phenotype (Hartman et al. 2001; Manolio et al. 2009).

Quantitative traits are not only one of the most important in the viewpoint of breeding programs but also can be influenced by a multiplicity of polymorphic genes, environmental conditions, and genetic interactions, making them extremely difficult to fully understand (Members of the Complex Trait Consortium 2003; Mackay 2014).

The purpose of the research reported in this article is to compare two methods of estimation of the parameter connected with the additive × additive × additive (*aaa*) interaction gene effect: the phenotypic method and the genotypic method. The comparison was made by analytical methods and with analyses of data sets of barley doubled haploid lines. To our knowledge, this is the first report about *aaa* interaction.

## Material and methods

If in the experiment we observed *n* homozygous (doubled haploid, DH; recombinant inbred, RI) plant lines, we get an *n*-vector of phenotypic mean observations ***y*** = [*y*_*1*_ *y*_*2*_ ... *y*_*n*_]’ and *q n*-vectors of marker genotype observations **m**_*l*_, *l* = 1, 2, …, *q*. The *i*-th element (*i* = 1, 2, …, *n*) of vector **m**_*l*_ is equal − 1 or 1, depending on the parent’s genotype exhibited by the *i*-th line.

### Estimation based on the phenotype

Estimation of the additive × additive × additive interaction of homozygous loci (three-way epistasis) effect *aaa* on the basis of phenotypic observations ***y*** requires identification of groups of extreme lines, i.e., lines with the minimal and maximal expression of the observed trait (Choo and Reinbergs 1982). The group of minimal lines consists of the lines which contain, theoretically, only alleles decreasing the value of the trait. Analogously, the group of maximal lines contains the lines which have only alleles increasing the trait value. In this paper, we identify the groups of extreme lines as minimal and maximal, respectively, lines of the empirical distribution of means. The total three-way epistasis interaction effect *aaa* can be estimated by the following formula:

1 $${\widehat{aaa}}_{p}=\frac{1}{2}\left({\overline{L} }_{\mathrm{max}}+{\overline{L} }_{min}\right)-\overline{L },$$

where ${\overline{L} }_{\mathrm{min}}$ and ${\overline{L} }_{\mathrm{max}}$ denote the means for the groups of minimal and maximal lines, respectively, $\overline{L }$ denotes the mean for all lines. The number of genes (number of effective factors) obtained on the basis of phenotypic observations only was calculated using the formula presented by Kaczmarek et al. (1988).

### Estimation based on the genotypic observations

Estimation of *aaa* is based on the assumption that the genes responsible for the trait are closely linked to the observed molecular marker. By choosing from all observed markers *p*, we can explain the variability of the trait, and model observations for the lines as follows:

2 $${\varvec{y}}={\varvec{1}\mu} +{\varvec{X}}{\varvec{\beta}}+{\varvec{Z}}{\varvec{\gamma}}+{\varvec{W}}{\varvec{\delta}}+{\varvec{e}},$$

where **1** denotes the *n*-dimensional vector of ones, *μ* denotes the general mean, ***X*** denotes *(n *× *p)*-dimensional matrix of the form $${\varvec{X}}=\left[\begin{array}{cc}\begin{array}{cc}{{\varvec{m}}}_{{l}_{1}}& {{\varvec{m}}}_{{l}_{2}}\end{array}& \begin{array}{cc}\cdots & {{\varvec{m}}}_{{l}_{p}}\end{array}\end{array}\right]$$, *l*_*1*_, *l*_*2*_, ..., *l*_*p*_ $\in$ {1, 2, ..., *q*}, ***β*** denotes the *p*-dimensional vector of unknown parameters of the form $${\varvec{\beta}}\boldsymbol{^{\prime}}=\left[\begin{array}{cc}\begin{array}{cc}{a}_{{l}_{1}}& {a}_{{l}_{2}}\end{array}& \begin{array}{cc}\cdots & {a}_{{l}_{p}}\end{array}\end{array}\right]$$, ***Z*** denotes matrix which columns are products of some columns of matrix ***X***, **γ** denotes the vector of unknown parameters of the form $${\varvec{\gamma}}\boldsymbol{^{\prime}}=\left[\begin{array}{cc}\begin{array}{cc}{aa}_{{l}_{1}{l}_{2}}& {aa}_{{{l}_{1}l}_{3}}\end{array}& \begin{array}{cc}\cdots & {aa}_{{{l}_{p-1}l}_{p}}\end{array}\end{array}\right]$$, ***W*** denotes matrix which columns are three-way products of some columns of matrix ***X***, *δ* denotes the vector of unknown parameters of the form $${\varvec{\delta}}\boldsymbol{^{\prime}}=\left[\begin{array}{cc}\begin{array}{cc}{aaa}_{{l}_{1}{l}_{2}{l}_{3}}& {aaa}_{{{l}_{1}{l}_{2}l}_{4}}\end{array}& \begin{array}{cc}\cdots & {aaa}_{{{{l}_{p-2}l}_{p-1}l}_{p}}\end{array}\end{array}\right]$$, and ***e*** denotes the *n*-dimensional vector of random variables such that *E(e*_*i*_*)* = 0, *Cov(e*_*i*_*, e*_*j*_*)* = 0 for *i* ≠ *j*, *i*, *j* = 1, 2, …, *n*. The parameters ${a}_{{l}_{1}}$, ${a}_{{l}_{2}}$, ..., ${a}_{{l}_{p}}$ are the additive effects of the genes controlling the trait, parameters ${aa}_{{l}_{1}{l}_{2}}$, ${aa}_{{l}_{1}{l}_{3}}$, ..., ${aa}_{{l}_{p-1}{l}_{p}}$ are the additive × additive interaction effects and parameters ${aaa}_{{l}_{1}{l}_{2}{l}_{3}}$, ${aaa}_{{l}_{1}{l}_{2}{l}_{4}}$, ..., ${aaa}_{{{l}_{p-2}l}_{p-1}{l}_{p}}$ are the additive × additive × additive interaction effects. We assume that the epistatic and three-way epistatic interaction effects show only loci with significant additive gene action effects. This assumption significantly decreases the number of potential significant effects and causes the regression model to be more useful.

Denoting by $\boldsymbol\alpha\boldsymbol'=\lbrack\mu\;\boldsymbol\beta\boldsymbol'\boldsymbol\;\boldsymbol\gamma\boldsymbol'\;\boldsymbol\delta\boldsymbol'\rbrack$ and $${\varvec{G}}=[\begin{array}{ccc}{\varvec1}& {\varvec{X}}& \begin{array}{cc}{\varvec{Z}}& {\varvec{W}}\end{array}\end{array}]$$ we obtain the model

3 $${\varvec{y}}={\varvec{G}}\boldsymbol{\alpha }+{\varvec{e}}.$$

If **G** is of full rank, the estimate of ${\varvec{\upalpha}}$ is given by (Searle 1982)

4 $$\widehat{\boldsymbol\alpha}={(\boldsymbol G'\boldsymbol G)}^{-1}\boldsymbol G\boldsymbol'\boldsymbol y\boldsymbol.$$

The total three-way epistasis *aaa* effect of genes influencing the trait can be found as follows:

5 $${\widehat{aaa}}_g={\textstyle\sum_{k=1}^{p-2}}{\textstyle\sum_{\underset{k'\neq k}{k'=2}}^{p-1}}{\textstyle\sum_{\underset{k''\neq k'}{k''=3}}^p}\;{\widehat{aaa}}_{l_kl_{k'}l_{k''}}.$$

For the marker selection of model (2), we used a stepwise feature selection by Akaike information criteria (Akaike 1998). The procedure consisted of two steps: first, we divided markers into groups based on chromosomes they were located on and performed stepwise feature selection by AIC; after that, we combined the remaining markers into one group and we repeated selection as above. All of the remaining markers were combined into the final group and the last feature selection was performed on a model with additive × additive × additive interaction effect included. To counteract the multiple comparisons problem, we used the Bonferroni correction.

### Examples

To compare the estimates of *aaa* obtained by different methods, the following data sets were used.

#### Example 1

The first set of data we used in our experiment comes from North American Barley Genome Mapping Project (NABGMP) and consists of 150 doubled haploid (DH) lines of barley tested in sixteen environments [Crookston, MN, 1992; Ithaca, NY, 1992; Guelph, Ontario, 1992; Pullman, WA, 1992; Brandon, Manitoba, 1992; Outlook, Saskatchewan, 1992; Goodale, Saskatchewan, 1992; Saskatoon, Saskatchewan, 1992; Tetonia, ID, 1992; Bozeman, MT (irrigated), 1992; Bozeman, MT (dryland), 1992; Aberdeen, ID, 1991; Klamath Falls, OR, 1991; Pullman, WA, 1991; Bozeman, MT (irrigated), 1991; and Bozeman, MT (dryland),1991]. Steptoe × Morex cross was developed by the Oregon State University Barley Breeding Program by crossing “Steptoe” and “Morex” barley varieties (Kleinhofs et al. 1993; Romagosa et al. 1996; http://wheat.pw.usda.gov/ggpages/SxM). The linkage map used consisted of 223 molecular markers, mostly RFLP, with mean distance between markers equal to 5.66 cM. Lines were analyzed for eight phenotypic traits (alpha amylase, AA; diastatic power, DP; grain protein, GP; grain yield, GY; height, H; heading date, HD; lodging, L; malt extract, ME; Hayes et al. 1993). Missing marker values were estimated with non-missing data of flanking markers (Martinez and Curnow 1994) and GP, L, and ME traits data were transformed by $arcsin\sqrt{x/100}$.

#### Example 2

The second data set also comes from the NABGM project and consist of 145 doubled haploid (DH) lines of barley (cross of two-rowed varieties Harrington × TR306) analyzed for seven phenotypic traits (weight of grain harvested per unit area, WG; number of days from planting until emergence of 50% of heads on main tillers, NH; number of days from planting until physiological maturity, NM; plant height, H; lodging transformed by $arcsin\sqrt{x/100}$, L; 1000 kernel weight, KW; test weight, TW) and tested in five environments (in four environments, observations were made over two years: Brandon, Manitoba, 1992 and 1993; Ailsa Craig, Ontario, 1992 and 1993; Elora, Ontario, 1992 and 1993; Outlook, Saskatchewan, 1992 and 1993; Ste-Anne-de-BeUevue, Quebec, 1993) (Tinker et al. 1996, http://wheat.pw.usda.gov/ggpages/HxT). We used the map composed of 127 molecular markers (mostly RFLP) with the mean distance between markers equal to 10.62 cm.

Considering that each trait and environment was classified as an independent variable in both cases, in total of 153 sets of observations were deemed. Trait data was transformed to achieve normal distribution of the observed features. In all cases, transformation was successful and normal distribution was obtained.

## Results

### Analytical comparison

The estimators, (1) and (5), of the three-way epistasis effect *aaa* can be analyzed and compared under simplified assumptions: (i) that the markers are unlinked and (ii) that the segregation of each marker is compatible with the genetic model appropriate for the analyzed population, which in our case means that the probability of observing “1” is the same as observing “ − 1”. This is true if we consider that model (2) treats the marker observations as fixed. In fact, the vectors **m**_*l*_, *l* = 1, 2, ..., *q*, constitute observations of some random variables. If the marker data satisfied exactly assumptions (i) and (ii) we would have

6 $${\widehat{aaa}}_g={\textstyle\sum_{k=1}^{p-2}}{\textstyle\sum_{\underset{k'\neq k}{k'=2}}^{p-1}}{\textstyle\sum_{\underset{k''\neq k'}{k''=3}}^p}\left[\frac12\left(\overline y^{(l_kl_k,l_{k''},+)}+\overline y^{(l_kl_k,l_{k''},-)}\right)-\overline y\right],$$

where $\overline y^{(l_kl_{k'}l_{k''},-)}$ and $\overline y^{(l_kl_{k'}l_{k''},+)}$ denote the means for lines with observations of *k*-th, *k’*-th, and *k’’*-th markers equal − 1 and 1, respectively.

In practice, the marker data do not accurately meet the following conditions for model (6). Taking into consideration that markers chosen for model (2) are far apart from each other on the linkage map, assumption (i) is true. To test the assumption (ii) ${\chi }^{2}$, the test is used before any analysis is performed.

### Numerical comparison

**Fig. 1:**
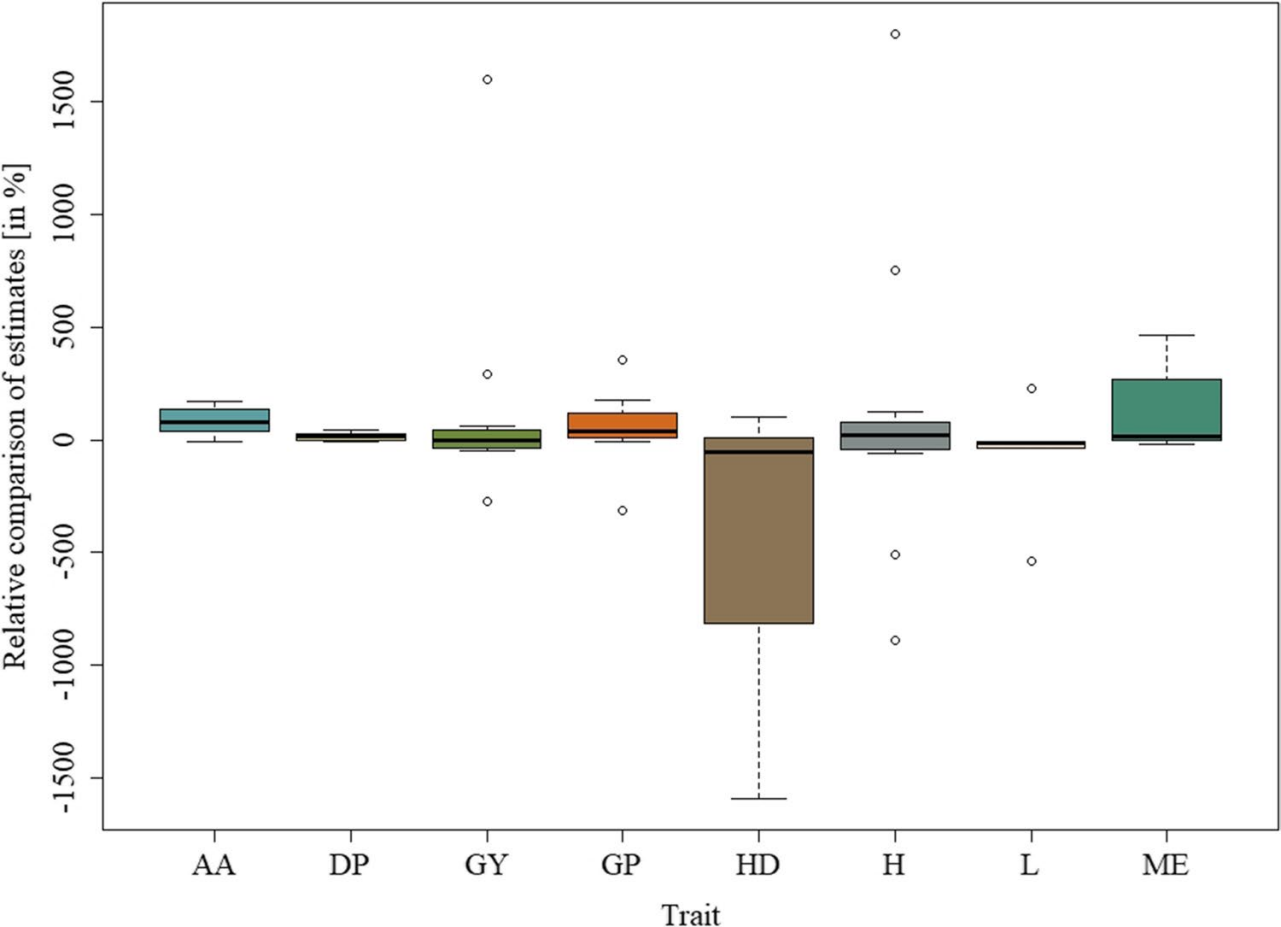
Relative comparison of phenotypic and genotypic estimates of the total additive × additive × additive interaction effect for the 150 doubled haploid lines of barley obtained from the Steptoe × Morex cross: box-and-whisker diagram of the values $\left(\widehat{{aaa}_{g}}/\widehat{{aaa}_{p}}\right)\bullet 100$, classified by the observed phenotypic traits (AA, alpha amylase; DP, diastatic power; GP, grain protein; GY, grain yield; H, height; HD, heading date; L, lodging; ME, malt extract)

**Fig. 2:**
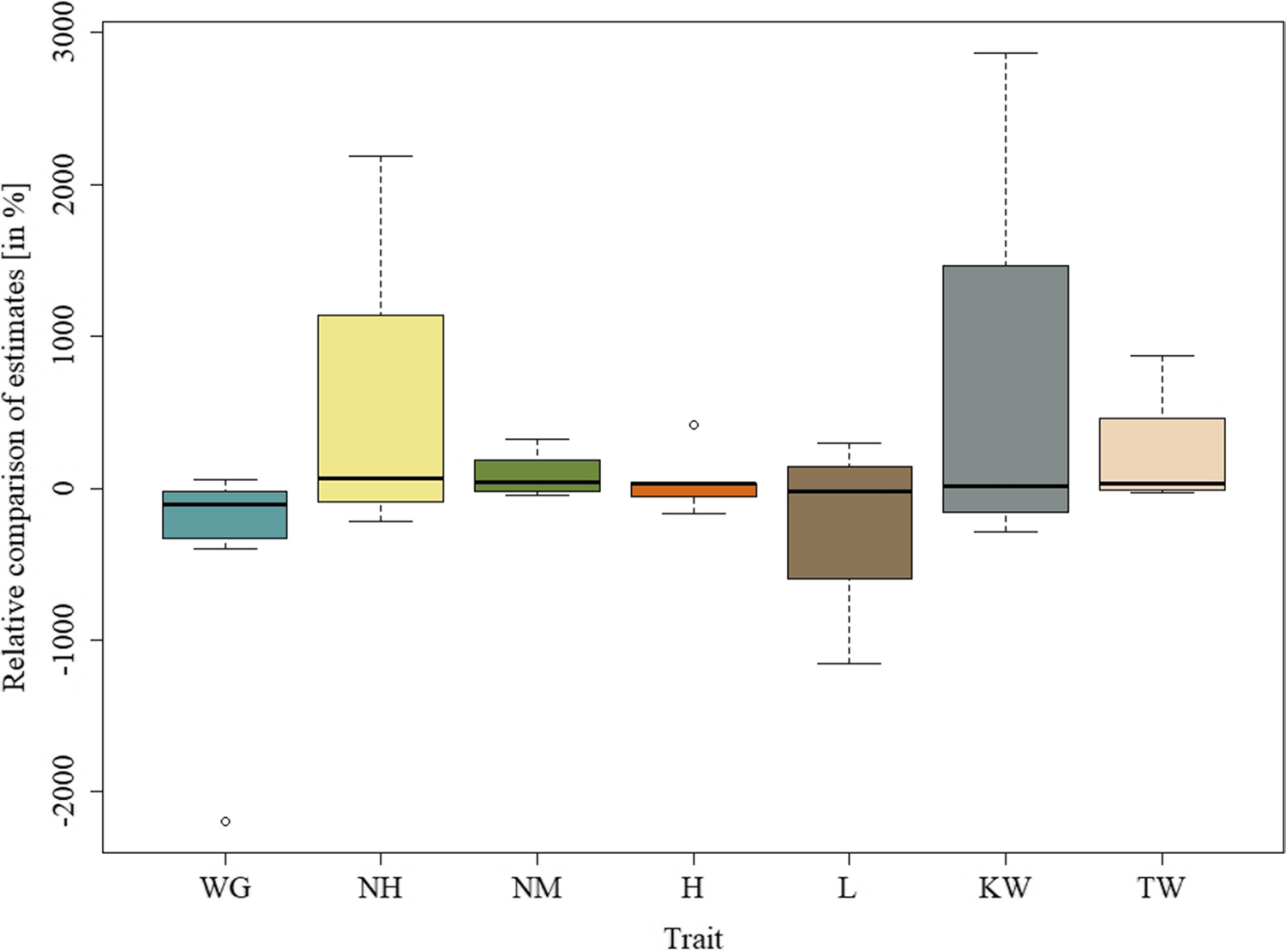
Relative comparison of phenotypic and genotypic estimates of the total additive × additive × additive interaction effect for the 145 doubled haploid lines of barley obtained from the cross Harrington × TR306: box-and-whisker diagram of the values $\left(\widehat{{aaa}_{g}}/\widehat{{aaa}_{p}}\right)\bullet 100$, classified by the observed phenotypic traits (H, plant height; KW, 1000 kernel weight; L, lodging; NH, number of days from planting until emergence of 50% of heads on main tillers; NM, number of days from planting until physiological maturity; TW, test weight; WG, weight of grain harvested per unit area)

Obtained results for estimates of total additive × additive × additive interaction effect was presented in Tables 1, 2, 3, and 4. Tables 1 and 2 contain phenotypic and genotypic analysis, respectively, for the 150 doubled haploid lines of barley from the Steptoe × Morex cross; Tables 3 and 4 for the 145 doubled haploid lines of barley from the Harrington × TR306 cross. Figures 1 and 2 show the relative comparison of phenotypic and genotypic estimates of the total additive × additive × additive interaction effect in the form of a box-and-whisker diagram of the values $\left(\widehat{{aaa}_{g}}/\widehat{{aaa}_{p}}\right)\bullet 100$, classified by the observed phenotypic traits.

**Table 1:** Phenotypic estimates of the total additive × additive × additive interaction effect for the 150 doubled haploid lines of barley obtained from the Steptoe × Morex cross

Environment	Trait							
	AA^$^	DP	GY	GP	HD	H	L	ME
ID91^#^	1.56 (5^@^)	11.36 (8)	− 0.31 (6)	0.08 (4)	− 0.05 (4)	0.90 (7)	-	0.45 (5)
ID92	1.72 (5)	31.17 (5)	− 0.78 (7)	0.21 (5)	− 0.75 (4)	6.42 (6)	-	0.37 (5)
MA92	-	-	− 0.22 (4)	-	− 0.79 (3)	− 0.48 (4)	3.14 (7)	-
MN92	3.13 (6)	5.16 (5)	− 0.54 (5)	0.24 (4)	0.93 (3)	1.33 (4)	-	0.61 (6)
MTd91	-	-	− 0.12 (4)	-	0.32 (3)	− 0.67 (5)	-	-
MTd92	2.82 (6)	28.11 (6)	− 0.74 (5)	0.50 (5)	0.68 (3)	6.25 (5)	14.50 (7)	− 0.33 (3)
MTi91	4.15 (4)	26.29 (10)	− 0.37 (3)	0.33 (5)	1.31 (4)	0.68 (5)	-	− 0.25 (5)
MTi92	1.03 (3)	20.32 (9)	− 0.13 (4)	1.30 (3)	− 0.73 (3)	3.55 (5)	4.93 (4)	− 0.73 (5)
NY92	-	-	0.26 (5)	-	0.31 (3)	− 1.33 (5)	20.87 (9)	-
ON92	-	-	− 0.14 (4)	-	0.98 (4)	1.03 (4)	11.80 (7)	-
OR91	3.82 (6)	17.59 (5)	0.54 (5)	0.46 (5)	− 2.66 (4)	− 4.64 (5)	-	− 0.10 (3)
SKg92	-	-	− 0.23 (4)	-	0.43 (4)	5.91 (4)	-	-
SKk92	-	-	− 0.34 (5)	-	0.55 (3)	0.55 (4)	-	-
SKo92	-	-	0.56 (6)	-	1.05 (3)	− 3.94 (5)	− 4.00 (3)	-
WA91	2.67 (5)	13.05 (4)	− 0.08 (4)	− 0.20 (5)	2.14 (5)	− 5.28 (5)	-	0.02 (6)
WA92	1.88 (4)	27.41 (9)	− 0.34 (6)	0.69 (3)	0.11 (4)	0.33 (3)	-	0.35 (4)

*ID91*, Aberdeen, ID, 1991; *ID92*, Tetonia, ID, 1992; *MA9*2, Brandon, Manitoba, 1992; *MN9*2, Crookston, MN, 1992; *MTd91*, Bozeman, MT, dry, 1991; *MTd92*, Bonzeman, MT, dry, 1992; *MTi91*, Bozeman, MT, irrigated, 1991; *MTi92*, Bozeman, MT, irrigated, 1992; *NY92*, Ithaca, NY, 1992; *ON92*, Guelph, Ontario, 1992; *OR91*, Klamath Falls, OR, 1991; *Kg9*2, Goodlae, Saskatchewan, 1992; *SKk9*2, Kcfr, Saskatchewan, 1992; *SKo9*2, Outlook, Saskatchewan, 1992; *WA91*, Pullman, WA, 1991; *WA92*, Pullman, WA, 1992. ^$^*AA*, alpha amylase; *DP*, diastatic power; *GP*, grain protein; *GY*, grain yield; *H*, height; *HD*, heading date; *L*, lodging; *ME*, malt extract. ^@^The number of genes (number of effective factors) obtained on the basis of phenotypic observations only

**Table 2:** Genotypic estimates of the total additive × additive × additive interaction effect for the 150 doubled haploid lines of barley obtained from the Steptoe × Morex cross

Environment	Trait							
	AA^$^	DP	GY	GP	HD	H	L	ME
ID91^#^	*NS **(17 | 0)	− 1.25 (32 | 1)	0.07 (22 | 8)	0.14 (17 | 25)	0.67 (20 | 11)	− 0.53 (24 | 10)	-	0.02 (27 | 1)
ID92	2.89 (23 | 13)	NS (14 | 0)	0.31 (22 | 9)	0.08 (21 | 1)	0.19 (27 | 3)	1.61 (25 | 5)	-	− 0.07 (27 | 3)
MA92	-	-	− 0.09 (18 | 1)	-	1.27 (20 | 16)	− 3.60 (20 | 10)	− 16.82 (15 | 23)	-
MN92	2.24 (26 | 2)	2.13 (19 | 17)	− 0.34 (18 | 7)	0.85 (16 | 23)	− 2.70 (19 | 23)	0.17 (17 | 20)	-	1.18 (22 | 14)
MTd91	-	-	0.33 (19 | 20)	-	0.06 (15 | 2)	3.41 (22 | 14)	-	-
MTd92	− 0.01 (29 | 2)	− 0.18 (24 | 6)	0.02 (20 | 12)	− 0.04 (27 | 4)	− 0.28 (24 | 4)	1.24 (23 | 4)	− 2.39 (26 | 6)	− 0.04 (31 | 1)
MTi91	− 0.25 (18 | 21)	NS (15 | 0)	− 1.08 (15 | 23)	0.08 (19 | 1)	NS (13 | 0)	NS (14 | 0)	-	NS (18 | 0)
MTi92	1.59 (18 | 7)	2.14 (16 | 28)	− 2.08 (20 | 16)	NS (13 | 0)	NS (12 | 0)	NS (18 | 0)	11.33 (21 | 11)	0.07 (15 | 2)
NY92	-	-	0.02 (22 | 10)	-	− 4.37 (18 | 25)	− 1.67 (16 | 21)	− 2.19 (22 | 6)	-
ON92	-	-	0.00 (25 | 7)	-	− 0.68 (25 | 4)	− 9.17 (20 | 11)	− 4.60 (24 | 13)	-
OR91	NS (15 | 0)	4.22 (15 | 5)	− 0.19 (15 | 1)	− 1.45 (17 | 30)	NS (15 | 0)	− 1.13 (16 | 1)	-	− 0.34 (22 | 9)
SKg92	-	-	NS (21 | 0)	-	NS (15 | 0)	− 1.46 (16 | 1)	-	-
SKk92	-	-	− 0.01 (16 | 4)	-	0.56 (16 | 30)	NS (17 | 0)	-	-
SKo92	-	-	− 0.13 (21 | 8)	-	0.37 (13 | 1)	− 1.32 (22 | 10)	0.44 (21 | 8)	-
WA91	3.20 (20 | 10)	NS (16 | 0)	NS (13 | 0)	− 0.12 (18 | 1)	0.07 (14 | 3)	NS (17 | 0)	-	NS (13 | 0)
WA92	1.44 (22 | 8)	3.88 (20 | 8)	0.16 (20 | 14)	0.25 (19 | 8)	− 1.75 (27 | 4)	5.94 (15 | 35)	-	1.63 (19 | 18)

^#^*ID91*, Aberdeen, ID, 1991; *ID92*, Tetonia, ID, 1992; *MA92*, Brandon, Manitoba, 1992; *MN92*, Crookston, MN, 1992; *MTd91*, Bozeman, MT, dry, 1991; *MTd92*, Bonzeman, MT, dry, 1992; *MTi91*, Bozeman, MT, irrigated, 1991; *MTi92*, Bozeman, MT, irrigated, 1992; *NY92*, Ithaca, NY, 1992; *ON9*2, Guelph, Ontario, 1992; *OR91*, Klamath Falls, OR, 1991; *Kg92*, Goodlae, Saskatchewan, 1992; *SKk92*, Kcfr, Saskatchewan, 1992; *SKo92*, Outlook, Saskatchewan, 1992; *WA91*, Pullman, WA, 1991; *WA92*, Pullman, WA, 1992. ^$^*AA*, alpha amylase; *DP*, diastatic power; *GP*, grain protein; *GY*, grain yield; *H*, height; *HD*, heading date; *L*, lodging; *ME*, malt extract. *NS, non significant; **(*x* | *y*): *x*, number of included markers, *y*, number of significant *aaa* interactions; “ − ”, *aaa* interaction not found

**Table 3:** Phenotypic estimates of the total additive × additive × additive interaction effect for the 145 doubled haploid lines of barley obtained from the cross Harrington × TR306

Environment	Trait						
	WG^$^	NH	NM	H	L	KW	TW
ON92a^#^	− 6.02 (10^@^)	0.11 (5)	− 1.34 (8)	1.87 (4)	9.24 (3)	1.28 (5)	− 0.77 (11)
ON93a	12.12 (7)	0.33 (6)	0.42 (8)	− 0.76 (9)	14.43 (3)	0.03 (6)	− 1.97 (2)
ON92b	6.21 (5)	0.27 (10)	0.08 (4)	0.26 (3)	− 0.34 (3)	0.81 (3)	− 0.60 (9)
ON93b	− 5.67 (7)	0.25 (6)	0.22 (3)	0.64 (6)	15.65 (6)	0.55 (5)	− 0.39 (8)
MB92	− 9.00 (5)	0.29 (9)	1.23 (11)	4.48 (4)	− 0.51 (4)	0.00 (8)	− 2.09 (3)
MB93	− 26.10 (6)	0.89 (11)	− 0.10 (4)	0.94 (9)	− 3.41 (8)	− 0.89 (6)	− 1.63 (13)
QC93	− 9.14 (5)	0.77 (7)	− 0.60 (3)	− 1.03 (3)	18.30 (5)	− 0.71 (3)	− 0.96 (5)
SK92a	61.75 (2)	1.15 (7)	0.12 (5)	1.78 (3)	− 9.58 (3)	− 2.11 (3)	− 2.93 (0)
SK93a	− 3.39 (7)	− 0.54 (4)	− 0.87 (5)	0.61 (3)	4.96 (2)	0.71 (7)	− 0.68 (8)

^#^*ON92a*, Ailsa Craig, Ontario, 1992; *ON93a*, Ailsa Craig, Ontario, 1993; *ON92b*, Elora, Ontario, 1992; *ON93b*, Elora, Ontario, 1993; *MB92*, Brandon, Manitoba, 1992; *MB93*, Brandon, Manitoba, 1993; *QC93*, Ste-Anne-de-Bellevue, Quebec, 1993; *SK92a*, Outlook, Saskatchewan, 1992; *SK93a*, Outlook, Saskatchewan, 1992. ^$^*WG*, weight of grain harvested per unit area; *NH*, number of days from planting until emergence of 50% of heads on main tillers; *NM*, number of days from planting until physiological maturity; *H*, plant height; *L*, lodging; *KW*, 1000 kernel weight; *TW*, test weight. ^@^The number of genes (number of effective factors) obtained on the basis of phenotypic observations only

**Table 4:** Genotypic estimates of the total additive × additive × additive interaction effect for the 145 doubled haploid lines of barley obtained from the cross Harrington × TR306

Environment	Trait						
	WG^$^	NH	NM	H	L	KW	TW
ON92a^#^	23.94 **(21 | 7)	NS (16 | 0)	− 0.34 (16 | 4)	0.56 (14 | 1)	NS (13 | 0)	0.72 (13 | 1)	− 0.46 (12 | 7)
ON93a	5.26 (16 | 1)	0.10 (12 | 1)	NS (15 | 0)	1.26 (15 | 1)	NS (12 | 0)	0.86 (13 | 1)	− 0.16 (7 | 1)
ON92b	− 5.54 (20 | 1)	NS (16 | 0)	0.15 (14 | 1)	NS (12 | 0)	3.93 (12 | 1)	− 0.21 (12 | 2)	− 5.21 (17 | 18)
ON93b	− 3.08 (13 | 1)	5.46 (16 | 15)	NS (9 | 0)	2.69 (16 | 26)	− 1.62 (15 | 21)	NS (15 | 0)	NS (13 | 0)
MB92	9.77 (14 | 1)	0.27 (13 | 4)	− 0.54 (14 | 3)	1.42 (14 | 2)	− 1.51 (13 | 1)	− 0.71 (11 | 2)	NS (16 | 0)
MB93	67.68 (14 | 36)	− 1.93 (14 | 24)	NS (14 | 0)	NS (10 | 0)	NS (12 | 0)	2.57 (18 | 12)	0.50 (15 | 3)
QC93	200.56 (17 | 20)	NS (13 | 0)	− 1.91 (15 | 30)	NS (13 | 0)	NS (16 | 0)	NS (13 | 0)	NS (11 | 0)
SK92a	*NS (7 | 0)	NS (17 | 0)	− 0.03 (12 | 2)	NS (11 | 0)	NS (13 | 0)	NS (12 | 0)	NS (16 | 0)
SK93a	NS (14 | 0)	NS (12 | 0)	− 0.40 (17 | 21)	− 0.34 (15 | 28)	− 1.46 (14 | 2)	NS (13 | 0)	NS (16 | 0)

^#^*ON92a*, Ailsa Craig, Ontario, 1992; *ON93a*, Ailsa Craig, Ontario, 1993; *ON92b*, Elora, Ontario, 1992; *ON93b*, Elora, Ontario, 1993; *MB92*, Brandon, Manitoba, 1992; *MB93*, Brandon, Manitoba, 1993; *QC93*, Ste-Anne-de-Bellevue, Quebec, 1993; *SK92a*, Outlook, Saskatchewan, 1992; *SK93a*, Outlook, Saskatchewan, 1992. ^$^*WG*, weight of grain harvested per unit area; *NH*, number of days from planting until emergence of 50% of heads on main tillers; *NM*, number of days from planting until physiological maturity; *H*, plant height; *L*, lodging; *KW*, 1000 kernel weight; *TW*, test weight. *NS, non significant; **(*x* | *y*): *x*, number of included markers, *y*, number of significant *aaa* interactions

Results show that in 90 cases (70%) we found statistically significant additive × additive × additive interaction effects (Table 1). The same amount of interactions was found for marker observation, but only in 72 cases, where we confirmed results statistically (Table 2). Comparisons of genotypic and phenotypic estimates of the total additive × additive × additive interaction effect show that in the majority of cases (79%), the effect was smaller than the total *aaa* interaction effect from phenotypic observations alone (Fig. 1). However, the scope of calculated estimates is quite large ranging from − 1590.91% for HD to 1800.00% for H in the same environment (WA92). In a total of five cases, we observed estimate values higher than |1000|%. The smallest range of estimates was observed for the trait DP. Number of genes (effective factors) ranged from 3–10 with average of 3.4 (Table 1). Minimal number of included markers equals 12, where maximum number was 32, with an average of 19.5 markers per model. The number of three-way interactions ranged from 0–35 with an average of 8.3 (Table 2).

For the Harrington × TR306, cross results show that in 63 cases (100%), we found statistically significant additive × additive × additive interaction effects (Table 3). The same amount of interactions was found for marker observation, but only in 35 cases, where we confirmed results statistically (Table 4). Comparisons of genotypic and phenotypic estimates of the total additive × additive × additive interaction effect show that in majority of cases (79%), the effect was smaller than the total *aaa* interaction effect from phenotypic observations alone (Fig. 2). Same as above, the scope of calculated estimates is quite large ranging from − 2194.31% for WG in environment QC93 to 2866.67% for KW in ON93a. In a total of four cases, we observed estimate values higher than |1000|%. The smallest range of estimates was observed for the trait NM. The number of genes (effective factors) ranged from 0–13 with an average of 5.6 (Table 3). A minimal number of included markers equals 7, where the maximum number was 21, with an average of 13.9 markers per model. The number of three-way interactions ranged from 0–36 with an average of 4.8 (Table 2).

In total, we analyzed 153 sets of observations, independently for each trait and each environment. Both examples were considered separately.

## Discussion

Breeding programs aim to enhance the most desirable traits. Actions based solely on phenotypic observations and gene effects are likely to miss the potentially huge impact of interaction and higher-order interaction effects (Taylor and Ehrenreich 2015). Analytical and numerical comparisons of methods of estimation of the total additive × additive × additive interaction effects are presented in this paper. The numerical comparison was conducted on 153 sets of observations from two examples of barley doubled haploid lines.

The analytic comparison shows that, under the assumption of correct segregation and no linkage between markers, the formulae for the phenotypic and genotypic estimators are comparable and that the additive × additive × additive interaction effect of each QTLs triad is smaller than the phenotypic effect.

The numerical comparison of estimates of additive × additive × additive interaction effect shows that in most cases (79% for both examples), genotypic estimate of *aaa* interaction is smaller than the phenotypic. This sentence is true due to the reason that phenotypic estimate consists of total additive × additive × additive interaction effects of all genes, unlike the genotypic estimate which includes only selected genes. For the rest of the cases that show lower values of phenotypic than genotypic estimates, it may be the result of a high genetic diversity with a lesser phenotypic diversity of the DH lines. High ranges of differences for the calculated estimates are most likely the result of a lot of different experimental variants such as different traits, environments, and experimental situations (Bocianowski and Krajewski 2009). The number of genes (effective factors) in phenotypic estimation does not directly influence the number of markers, as well as the number of *aaa* interaction included in genotypic models. Both the number of effective factors and number of markers are pretty consistent with few outliers, which makes sense considering that our method tries to include the maximum amount of best-fitted factors. On the contrary, the number of *aaa* interactions ranged quite widely which may be the result of omitting markers that by themselves do not improve the model but can create the best threes.

In this paper, stepwise feature selection by Akaike information criteria was used. We received comparable results to the previous paper using the same datasets (Bocianowski 2012) with backward stepwise regression as well as to the method of inclusive interval mapping (ICIM) (described by Li et al. 2008). The presented results show that the inclusion of higher-order (*aaa*) interactions in multiple regression models can have an exert influence on QTL effect.

An important assumption to make is that *aaa* interaction effects show only loci connected to markers with significant effects. Including additional markers may reveal additional interaction but with significant increase of data quantity requirement (Manolio et al. 2009). Further studies are necessary with respect to additive × additive × additive interaction effects conducted by machine learning methods and by simulation analysis that would make possible consideration of different experimental situations. Current data was not sufficient enough to use machine learning for feature selection. For data containing more markers, we suggest the use of LASSO and SHAP values methods.

## Conclusions

Higher-order interactions are usually neglected due to extensive data requirements, although this does not mean they are irrelevant, on the contrary —— higher-order interactions occur often and can have a huge impact on phenotype.

The presented methods were useful statistical tools for QTL characteristics and allow estimating *aaa* interactions.

On the basis of available literature, this is the first report concerning the presence of analytical and numerical comparisons of two methods of estimation of additive × additive × additive interaction of QTL effects.

Further studies of higher-order interactions and methods of their estimation are necessary.

## Data Availability

The data presented in this study are available on request from the corresponding authors.
